# Retraction: Dysregulation of Fra1 expression by Wnt/β-catenin signalling promotes glioma aggressiveness through epithelial–mesenchymal transition

**DOI:** 10.1042/BSR20160643_RET

**Published:** 2026-05-13

**Authors:** Huaijun Liu, Li Zhang, Xiaodan Mu, Jianling Cui, Zhigang Peng

**Affiliations:** Department of Medical Imaging, The Second Hospital of Hebei Medical University, Shijiazhuang 050000, Hebei Province, China

**Keywords:** β-catenin, EMT, Fra1, glioma, Wnt

This article is being retracted from *Bioscience Reports* at the request of the Editor-in-Chief and the Editorial Board.

This follows the identification of concerns involving Figure 3 of the article, alerting the Editorial Board to similarities between the images in Figure 3B, F and I, when compared Figures in the articles Su et al. 2019 (DOI: 10.1186/s11658-019-0149-x) and Wang et al. 2017 (DOI: 10.3892/ijo.2017.4165).

Specific similarities between this article and Su et al. 2019 (DOI: 10.1186/s11658-019-0149-x) include:

- Between the Figure 3F +/+ Transwell panel from this article and Figure 2A U87MG +/-/+ panel of Su et al. (2019)

Specific similarities between this article and Wang et al. 2017 (DOI: 10.3892/ijo.2017.4165) include:

- Between the Figure 3B siCtrl Transwell panel of this article and the Figure 1E shE2F1 panel of Wang et al. (2017)
- Between the Figure 3F +/+ Transwell panel of this article and the Figure 4D EV shE2F1 panel of Wang et al. (2017)
- Between the Figure 3F -/- Transwell panel of this article and the Figure 4D EV shE2F1 panel of Wang et al. (2017)
- Between the Figure 3F +/- Transwell panel of this article and the Figure 4D DDR1 shCtrl panel of Wang et al. (2017)
- Between the western blots of Figure 3I of this article and the Figure 5A blots from Wang et al. (2017); an example being the Figure 3I Zeb1 blots of this article and the Figure 5A E-cad blots from Wang et al. (2017).

The authors have been contacted with regards to the retraction, but they have not responded to the Journal's queries or the concerns raised. Given the extent of the issues raised, the Editorial Board stands by the decision to retract the article.

